# Invasion Status, Mechanisms, and Future Distribution Prediction of *Solidago canadensis* in the Trade Port Region: A Case Study of Ningbo Port, China

**DOI:** 10.3390/plants14101546

**Published:** 2025-05-21

**Authors:** Xu Luo, Sixiao Shen, Ke Liao, Saiqiang Li, Qinqin Pan, Jiahao Ma, Weiqiang Li, Xiaodong Yang

**Affiliations:** 1 Ningbo University Donghai Academy, Zhejiang Ocean Development Think Tank Alliance, Ningbo 315211, China; 2211420021@nbu.edu.cn (S.S.); 2111073012@nbu.edu.cn (K.L.); 2211420010@nbu.edu.cn (S.L.); 2211420052@nbu.edu.cn (Q.P.); 2311110180@nbu.edu.cn (J.M.); 2311110174@nbu.edu.cn (W.L.); 2 Department of Geography & Spatial Information Techniques, Ningbo University, Ningbo 315211, China

**Keywords:** invasive alien species, distribution patterns, ecological risk, species distribution model, future climate scenarios

## Abstract

Trade ports are the first places where alien species invade and the source of their spread to other areas. Controlling invasions in these regions can effectively reduce invasion pressure and disrupt the spread pathways of invasive species, thereby significantly reducing their threat to local ecosystems and biodiversity loss. Based on 595 field survey plots, the Generalized Linear Model (GLM) and Species Distribution Model (MaxEnt) were employed to analyze and predict the invasion mechanisms and future possible distribution of *Solidago canadensis* in the Ningbo Port, China. The results indicate that the invasion of *S. canadensis* in the Ningbo Port was particularly severe, with a 67.7% occurrence rate of all sampling plots in the field survey, and a risk level classified as Grade 1. Biodiversity (*p* < 0.001) and the minimum temperature of the coldest month (*p* < 0.01) significantly affect the invasiveness. Highly diverse communities could resist the invasion of alien species, which support Elton’s diversity–invasibility hypothesis. Low temperatures had a restrictive effect on the invasion of *S. canadensis*. The total suitable area continued to expand under three different climate change scenarios compared to current conditions (increased by 3.73%, 5.67%, and 3.74% by the 2070s). The total potential habitat area of *S. canadensis* reached its maximum extent (89.77%) under the medium greenhouse gas emission scenario in the 2050s. Meanwhile, the medium suitable area exhibited the greatest fluctuation among the three climate scenarios. Under the low emission condition, the medium suitable area of *S. canadensis* diminished by 63.10 km^2^, but in the medium and high emission condition, its area expanded by 91.13 km^2^ and 16.20 km^2^, respectively. Under future climate warming scenarios, the invasion risk of *S. canadensis* in Ningbo Port will continue to increase. The results of our study reveal the diffusion mechanisms of invasive plants at the colonization source, providing important theoretical support for invasive alien species’ initial prevention and control.

## 1. Introduction

Influenced by global trade and climate change, invasive alien species (IAS) have become a significant threat to global ecological security [1,2]. Taking China as an example, the number of IAS increased from 126 in 2005 to 403 in 2020 [3], causing economic losses of approximately USD 14.45 billion, which severely threatens ecological security, biodiversity, and food security [4]. As the core nodes of global trade, ports handle over 80% of the world’s cargo volume and serve as major hubs for trade and maritime traffic [5]. The frequent ship transportation and human activities in the port region make these regions the first to be colonized by invasive species, and the richness of alien plants in these regions is obviously higher than that in near-natural ecosystems [6]. The research of 54 waterway ports in Central Europe over the past 40 years has shown that alien species account for 41% of all species and increase with the decrease in the distance from the ocean [7]. Moreover, studies shown a positive correlation between the first record of alien species and international import volume [8]. It was projected that, by 2050, the global shipping volume will further increase by 240% to 1209%, leading to significant changes in the risk of alien species invasion in port areas [9]. Thus, effectively controlling IAS in the port region plays a crucial role in preventing their wide spread and in formulating control measures.

The successful establishment of IAS is influenced by factors such as species characteristics, community diversity, climate, and soil nutrition [10,11,12,13]. Studies have demonstrated that variations in species traits play a crucial role in determining the success of invasions [12,14]. For example, *Conyza canadensis* successfully invaded eastern China due to its superior light acquisition ability compared to local species [15]. *Ailanthus altissima* successfully invaded all administrative regions in Italy through competition [16]. Moreover, some studies using comparative methods have found that many IAS, such as *Ageratina adenophora* and *Jacobaea vulgaris*, enhance the probability of their successful invasion by adjusting the nutrient distribution and functional traits of leaves [17,18,19]. The Elton’s diversity–invasibility hypothesis posits that a higher species diversity in local communities reduces the likelihood of successful invasion by alien plants [20]. The successful establishment of IAS is also closely related to environmental conditions. For instance, Dawson et al. [13] found that abundant resources facilitate biological invasions. However, in contrast, Funk et al. [21] concluded that areas with resource scarcity can also be successfully invaded because IAS have a higher resource utilization efficiency than local species in resource-poor environments. Most existing studies primarily focus on analyzing community survey data post-invasion, aiming to uncover the relationship among invasion intensity, diversity, and environmental factors to explain invasion mechanisms [22,23,24]. They relatively lack analyses of the colonization source, specifically in the trade port region where alien species first arrive. This limitation hinders the implementation of control measures at the source.

*Solidago canadensis* is a perennial herb that belongs to the *Asteraceae* family, indigenous to the United States and Canada [25]. Initially imported to China in 1935 as an ornamental species, it subsequently escaped cultivation and became an invasive problem [26]. *S. canadensis* has a strong competitive ability due to its tall, rapid growth and high reproductive capacity [15,27], and can quickly form monodominant stands in invaded areas, severely threatening local biodiversity and ecological balance [28]. Moreover, it exhibits strong allelopathic effects, as its root exudates can inhibit the growth and development of various crops [29], resulting in reduced crop yields and quality decline. Thus, understanding the invasion mechanisms of *S. canadensis* and seeking control methods have become a hot issue for local governments and the academic community.

Ningbo Port is the largest foreign trade port in the world by cargo throughput, with approximately 25% of its containers originating from North America, which is the native region of *S. canadensis* [30]. The reproductive bodies of this species are spread through containers, ballast water, or transport vehicles along roads, rivers, and other pathways [31,32]. Among them, ballast water alone can transfer 3000 to 4000 different species every day [33]. Furthermore, a large number of artificial structures in port regions (such as docks and breakwaters) provide important spatial resources for the settlement and spread of IAS [34]. Thus, port regions are highly vulnerable to invasion and the establishment of IAS. Our study investigated the current invasion status of *S. canadensis* in the Ningbo Port region, analyzed the influencing factors, and aimed to reveal the invasion mechanisms and predict its invasion trends under different future climate scenarios. The specific objectives of this study were: (1) to analyze the current geographic distribution and invasion risk level of *S. canadensis* in Ningbo Port; (2) to reveal its invasion mechanisms in port regions; and (3) to predict the trend of its potential distribution area in the port region under different climate scenarios in the future.

## 2. Results

### 2.1. Invasion Status and Risk Level

**Figure 1:**
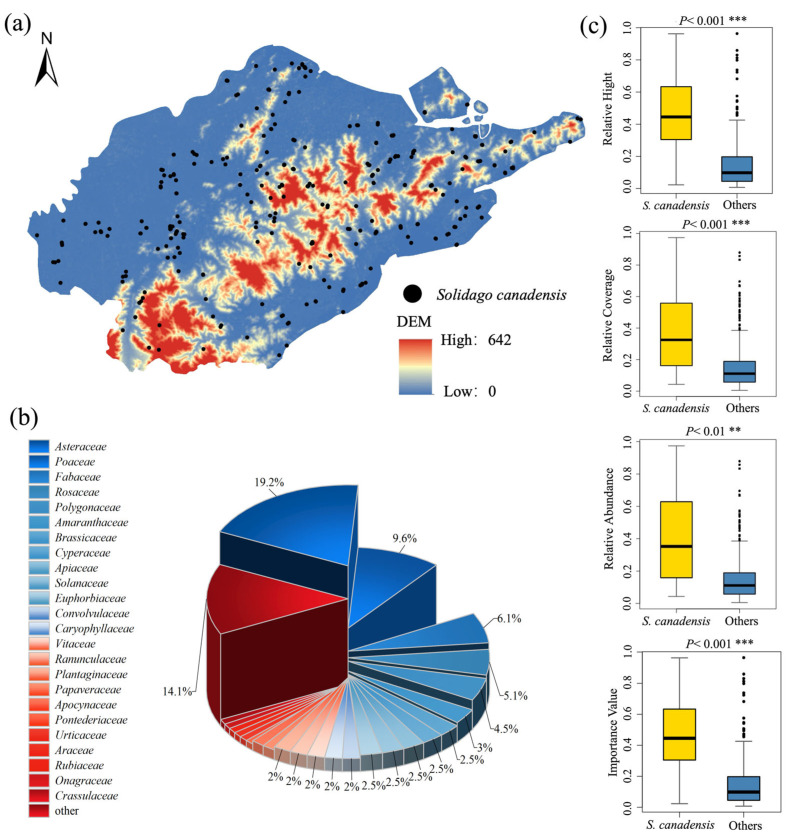
Invasion status of *S. canadensis* in Ningbo Port. (**a**) The distribution map of *S. canadensis*; (**b**) showed the proportion of plant families, with the lower part of the family (<1.0%) being merged into the others; and (**c**) presented the T test results for *S. canadensis* compared to other species.

We surveyed a total of 205 species in 595 sample plots; these species belong to 53 families, with the *Asteraceae* family having the highest proportion (19.2%) (Figure 1b). The next were *Poaceae and Fabaceae*, accounting for 9.6% and 6.1%, respectively. *S. canadensis* was widely distributed in the study area, exhibiting a scattered multi-point distribution pattern. *S. canadensis* was present in 403 of the total plots, accounting for 67.7% (Figure 1a). In addition, *S. canadensis* held a competitive advantage in the plots, with mean values of relative height, relative cover, relative abundance, and ecological importance of 0.459, 0.379, 0.381, and 0.457, respectively. Other species had a more dispersed distribution of height, coverage, abundance, and importance value. All values of *S. canadensis* were significantly higher than those of other species for relative height (*p* < 0.001), relative cover (*p* < 0.001), relative abundance (*p* < 0.01), and importance (*p* < 0.001) (Figure 1c). Among them, the plant height showed the most significant difference.

Based on the formulas mentioned in the Section 4.2 and Table S1 (list in the Supplementary Materials), the risk assessment of *S. canadensis* indicated an evaluation score of 2.92, classifying it as a level 1 invasion risk. This was consistent with the classification in the Chinese Alien Invasive Species Information System (https://www.iplant.cn/ias/, accessed on 13 May 2025), where it was categorized as a malicious invader.

### 2.2. The Relationship Between Invasion Intensity, Plant Diversity, and Environmental Factors

**Figure 2:**
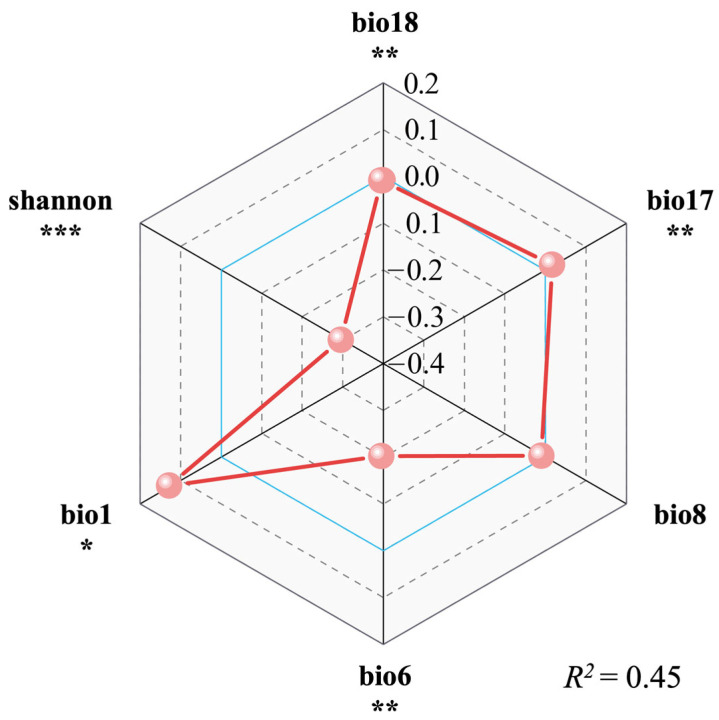
The relationship of IAS ecological importance to species diversity and environmental factors. *** *p* < 0.001, ** *p* < 0.01, and * *p* < 0.05.

We analyzed the selected variables using the GLM. The *R*^2^ of the model was 0.45, indicated a relatively good explanatory power. The results of GLM show that the Shannon–Wiener Diversity Index was the most important factor on the invasiveness of *S. canadensis*, and there was an extremely significant negative correlation between them (*p* < 0.001). Among the variables, bio6 and bio18 showed significant negative correlations with the invasiveness of *S. canadensis* (*p* < 0.01). Notably, bio6 contributes more than bio18 to this relationship. Oppositely, it had a significant positive correlation with bio1 (*p* < 0.01) and bio17 (*p* < 0.01). Although a negative correlation between invasiveness and bio8 was observed, it was not statistically significant (Figure 2).

### 2.3. Potential Distribution of S. canadensis Under Climate Scenarios

**Figure 3:**
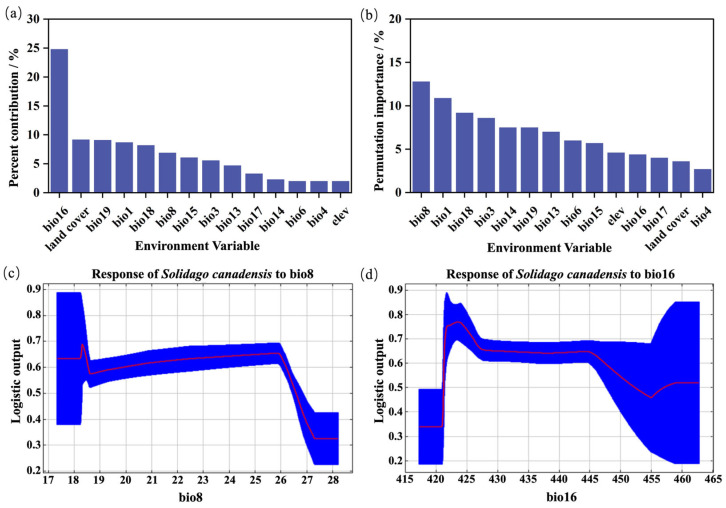
Response curves of high–weight influencing factors for *S. canadensis* under current climate conditions. (**a**,**b**) represent the percent contribution and permutation importance of environmental factors to *S. canadensis*, respectively. (**c**,**d**) respectively represent the response curves of the most influential environmental variables for *S. canadensis*.

The AUC value of this model was 0.845, which indicates that the performance of the model had relatively good predictive accuracy (Figure S1). The percentage contribution of each environmental factors is shown in Figure 3a, with bio16 having the highest contribution (26.1%), followed by land cover (9.7%), bio19 (9.6%), bio1 (9.2%), bio18 (8.6%), bio8 (7.3%), bio15 (6.4%), and bio3 (5.9%). The total contribution of these factors was 82.7%. The permutation importance of the environmental factors is shown in Figure 3b, with bio18 having the highest importance (12.1%), followed by bio1 (11.2%), bio18 (9.2%), bio3 (8.6%), bio19 (7.5%), bio14 (7.5%), bio13 (7.0%), and bio6 (6.0%). After reclassification, the total suitable area of *S. canadensis* under current climate conditions was estimated: 244.24 km^2^ in the low suitable areas, 458.88 km^2^ in the medium suitable areas, and 240.55 km^2^ in the highly suitable areas, with the total suitable habitat area accounting for 84.10%.

Single-factor modeling was performed for bio8 and bio16. The results show that the probability of *S. canadensis* distribution fluctuated significantly with changes in the environmental variables. When bio8 was between 17 and 18 °C, the probability of *S. canadensis* presence remained stable at 0.64 (Figure 3c). As the temperature increased to 18.2~18.5 °C, the probability gradually increased, reaching a peak value of 0.69. It was stable within the range of 18.6 °C~25.9 °C. When the temperature exceeded 25.9 °C, the distribution probability decreased rapidly and stabilized at 0.33 within the range of 27.3~28.1 °C. When bio16 was between 417 and 421 mm, the probability of *S. canadensis* distribution was 0.33 (Figure 3d). As the precipitation increased to 421~424 mm, the probability gradually rose, peaking at 0.76, stably existing in the range of 427~445 mm. When the precipitation exceeded 424 mm, the distribution probability showed a decreasing trend, stabilizing at 0.51 between 458 and 464 mm.

**Figure 4:**
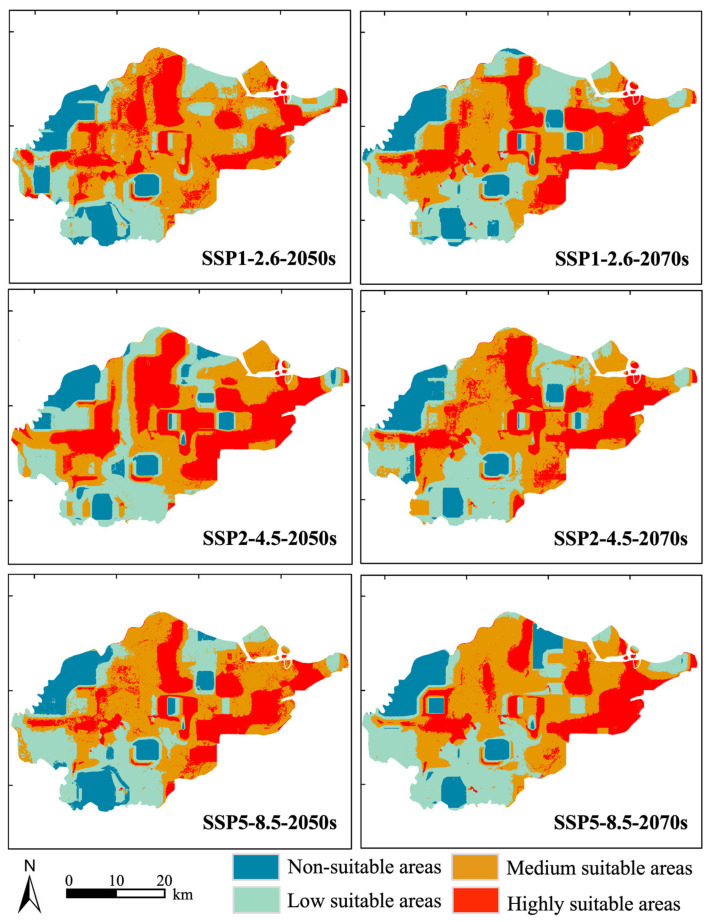
Potential geographic distribution of *S. canadensis* under different future climate change scenarios.

Under all SSP scenarios, the total suitable habitat area for *S. canadensis* was higher than that under current climate conditions, although the increase was not substantial (Figure 4). The highly suitable habitat area had a relatively wide distribution, mainly concentrated in the five major port regions of Ningbo Port, extending inland. Under low greenhouse gas emission conditions and medium greenhouse gas emission conditions (from 2050s to 2070s), the percentage of total suitable habitat area decreased from 88.12% to 87.83% and from 89.77% to 89.46%, respectively. Under high greenhouse gas emission conditions, the proportion of total suitable habitat area increased from 86.39% to 87.84%. Specifically, the highly suitable habitat area increased by 0.66 km^2^, the medium suitable area decreased by 63.10 km^2^, and the low suitable area increased by 59.09 km^2^ under low emission greenhouse gas conditions. Under medium greenhouse gas emission conditions, the highly suitable area decreased by 84.03 km^2^, the medium suitable area increased by 91.13 km^2^, and the low suitable habitat area decreased by 10.55 km^2^. Under high greenhouse gas emission conditions, the highly suitable area decreased by 25.17 km^2^, the medium suitable area increased by 16.20 km^2^, and the low suitable area increased by 25.25 km^2^. In summary, under scenarios of low greenhouse gas emissions and medium greenhouse gas emissions, the total suitable habitat area for *S. canadensis* reached its maximum proportion in the 2050s and then declined slowly. However, under high greenhouse gas emission conditions, the total suitable habitat area continued to increase.

## 3. Discussion

### 3.1. The Invasion of S. canadensis Was Severe in the Port Region

Our field survey of *S. canadensis* in Ningbo Port showed that *S. canadensis* was present in 403 out of 595 plots, accounting for 67.73%, indicating a severe invasion. This was due to the long history of *S. canadensis* invasion in the area. *S. canadensis* first escaped to Zhejiang and Jiangsu provinces after entering Shanghai city [35]. Furthermore, ports were the first areas where *S. canadensis* established itself through transportation routes. Ningbo Port, with its numerous inner and outer ports and frequent human activity, facilitated the efficient spread of the species via rail, road, and water transport [2]. *S. canadensis* reproduces sexually and can be wind-dispersed, with a single plant producing 15,000–25,000 seeds annually. The higher average wind speed in coastal cities accelerated seed dispersal [36]. Our results show that *S. canadensis* had significant advantages in its traits. Its height usually ranges from 1 to 3 m, which is quite different from that of other local species. This made it more competitive in the struggle for resources and enabled it to become an advantageous species. Meanwhile, the competitive advantage in abundance and cover enhanced shading within the community, led to vegetation homogenization, and reduced community diversity [37,38]. The study conducted by Wang et al. further demonstrated that the height of *S. canadensis* was a critical factor at all stages of invasion, as it occupied distinct spatial niches compared to local species [15,39].

The risk assessment of the invasion of *S. canadensis* showed it as level 1, and it is regarded as a malicious invader. This classification indicated that *S. canadensis* poses a high ecological threat and had a significant dispersal potential in the Ningbo Port region. As a malicious IAS, its high reproductive capacity, broad adaptability, and allelopathic effects enabled it to rapidly occupy local ecological niches, substantially inhibiting the establishment and development of native plant community, which resulted in biodiversity decline and impairment in ecosystem functions [29,40]. Our results indicate the severity of the invasion risk posed by *S. canadensis*. Therefore, regulatory authorities should enhance monitoring and management in port trade areas to reduce the spread of invasive alien plants.

### 3.2. The Major Underlying Drivers of S. canadensis Invasion

Our results show a significant negative relationship between the invasiveness of *S. canadensis* and species diversity within the community, which was highly consistent with Elton’s diversity–invasibility hypothesis [20]. Native species occupy multidimensional ecological niches, reducing resource availability to resist alien species. This mechanism was also confirmed in experimental studies on IAS such as *Phragmites australis*, *Senecio*, and *Cytisus scoparius* [41,42]. Furthermore, the mixed allelopathic substances in high-diversity communities can counteract the singular allelopathic effects of *S. canadensis*, weakening its suppression of the growth and development of local species [35].

Climate change influences multiple mechanisms underlying the success of biological invasions [43], with temperature playing a particularly significant role [44]. Our results show that the invasion intensity of *S. canadensis* in Ningbo Port decreased with a lower minimum temperature of the coldest month. The possible reason is that *S. canadensis* is a temperate plant, and changes in climate niches are relatively rare among terrestrial invasive plants [45]. Thus, the low-temperature limitation threshold of *S. canadensis* suppressed its invasion, while native plants were unaffected [44]. Another major negative influencing factor was bio18. It may be that *S. canadensis* is better adapted to drier or moderately humid environments, while excessive rainfall could potentially affect root respiration and inhibit its invasion. These two negative influencing factors may suggest that the success of *S. canadensis* invasion depends on specific climatic windows. The increase in bio1 shortens the plant growth cycle, which may improve the overwinter survival rate of *S. canadensis* and its seeds [46,47], while bio17 may suppress local plants (such as annual herbs), releasing their ecological niche. The effects of these two factors alleviate the competitive pressure on *S. canadensis*, enhancing its invasion intensity within the community.

### 3.3. The Potential Area of S. canadensis Under Different Climate Scenarios

Under the current climate scenario, *S. canadensis* exhibited strong adaptability in the Ningbo Port region. The total area of suitable habitat accounted for 84.1%, primarily consisting of medium and highly suitable zones, indicating elevated invasion pressure. The response curve under the current climate conditions showed that *S. canadensis* had a relatively long suitable growth range in the Ningbo Port region. Under the context of global warming, the total suitable habitat area for *S. canadensis* was higher than that under the current climate scenario, which may be due to its preference for moderately warmer and wetter climatic conditions. The increasing trend under future high emission climate scenarios was evident. This indicates that the invasion of *S. canadensis* in the Ningbo Port has not yet reached saturation, and its invasion risk is rising, suggesting a potential outbreak. This result aligns with the findings of Seebens et al. [48]. Spatially, the potential highly suitable habitat was relatively dispersed in all three climate scenarios and was mainly located near five major ports. Among them, Meishan Port and its surrounding areas in the southeastern part showed the most concentration. This was because ports, as sources of invasion, were the initial areas where *S. canadensis* established itself and spread rapidly. Ports acted as “stepping stones” for alien species to invade inland areas, and studies in Taiwan and Hainan have also highlighted the serious issue of alien species invasions in ports [49,50]. The changes in various suitable habitat zones within the port region under the three climatic scenarios differed. The reason may be related to the sensitivity of *S. canadensis* to dynamic changes in temperature and precipitation, with Ningbo Port being mostly coastal lowlands, with a humid climate and significant influence from monsoons and typhoons. Medium suitable habitat areas may be concentrated in the transition zones from the ports to the inland (such as farmland and wetland edges), where *S. canadensis* is more sensitive to changes in temperature and precipitation. The response curve of high–weight factors also reflected that *S. canadensis* prefers warm conditions but not excessive heat. Therefore, regulatory authorities should strengthen the prevention, monitoring, and management of IAS in the port region. Control measures should be prioritized based on the suitability of habitat areas, particularly for highly suitable and medium suitable areas. A multi-scale, cross-regional monitoring network should be established using satellite remote sensing and drone patrols. Additionally, physical removal should be carried out during the growth window of *S. canadensis*, such as before flowering and seeding or during the coldest month. Its spread could also be mitigated by increasing local species diversity.

Our study mainly focused on exploring the impact of climate change on the distribution of IAS, with less consideration of human activities. Meanwhile, the optimal model we adopted only considered the land cover factor, which might be inadequate. Because of the frequent human activities in port regions, the propagules can be spread through cargo transportation, vehicle tires, personnel clothing, and other ways. Therefore, future studies should consider more factors related to human activities (such as traffic density, land use intensity etc.) to better understand their impact on IAS distribution patterns. In addition to Elton’s diversity–invasibility hypothesis, the current theories about the invasion mechanism of alien species also include the Darwin puzzle (naturalization hypothesis and preadaptation hypothesis). However, we did not analyze genetic diversity or niche overlap to validate these theories, which may limit the generality and scalability of our study. Furthermore, the invasion mechanisms at trade ports in different regions also vary. Future research should expand the scale of study, implement long-term monitoring, and comprehensively consider various influencing factors. These future studies are conducive to a more comprehensive understanding of the contributions and applicability of different invasion mechanisms. This will provide stronger theoretical support for managing IAS, protecting ecosystems, and preserving biodiversity.

## 4. Materials and Methods

### 4.1. Study Area

**Figure 5:**
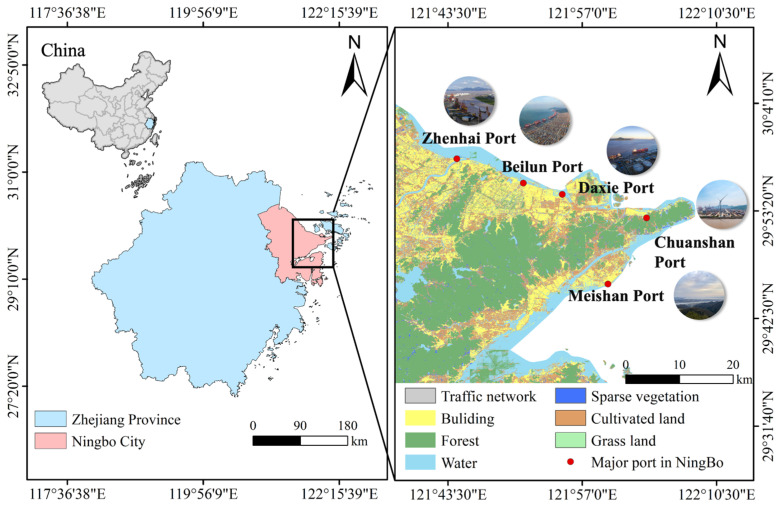
Map of the study area.

Ningbo Port is the port with the highest number of large and extra-large deep-water berths in mainland China. As of the end of 2023, it has 205 large deep-water berths of over 1 × 10^4^ tons and 129 extra-large deep-water berths of over 5 × 10^4^ tons (data from the Ningbo Port’s official website (www.nbport.com.cn, accessed on 8 April 2025)). It consists of several port regions, including Beilun, Zhenhai, Ningbo, Daxie, Chuanshan North, and Meishan. Ningbo Port is located in Ningbo city, in southeastern China, covering a total land area of 9816 km^2^ (Figure 5) [51]. The climate type is characterized by subtropical monsoon climate, with 22 days of high temperatures, 18 days of frost, an average annual temperature of 17.4 °C, 1850 h of annual sunshine, and annual precipitation of 1480 mm [52,53]. The local vegetation belongs to the northern subzone of the central subtropical evergreen broadleaf forest, with dominant woody plants such as *Fagaceae* and *Lauraceae* [54].

### 4.2. Methods

#### 4.2.1. Field Community Surveys

Through the reviewing of the literature, the distribution status of IAS in the Ningbo Port and its surrounding areas was initially understood, and potential distribution areas were identified (Figure 5). Comprehensive field surveys were conducted in these potential areas. Surveys were conducted during the plant growing seasons from 2022 to 2023. The survey habitats included forests, wetlands, and grasslands. A simple random sampling method was used to establish 1 m × 1 m herbaceous plots along different survey routes within the study area, totaling 595 plots. We recorded the coordinates (latitude and longitude) and elevation and conducted a community survey for all plants within the plots. The survey indicators included species names, abundance, heights, and coverage. The types of communities in the study area are shown in Table 1.

#### 4.2.2. Environmental Data

The environmental data used in this study included climate data, soil data, and topographic data. Current climate condition data were obtained from 6 meteorological observation stations provided by the local meteorological department, consisting of a total of 19 variables (Table 2). We selected future climate data from three climate change scenarios (SSP1-2.6, SSP2-4.5, SSP5-8.5), with low greenhouse gas emission, medium greenhouse gas emissions, and high greenhouse gas emissions under the BCC-CSM2-MR model for prediction, with a resolution of 2.5 arc-minutes for all scenarios. The soil data included soil salinity, moisture content, and pH. These were determined through laboratory experiments. Specifically, we collected 0–20 cm of topsoil in each quadrat center and conducted laboratory experiments after completing community surveys. The topographic data were sourced from the Geospatial Data Cloud, with a resolution of 30 m × 30 m.

#### 4.2.3. Risk Assessment of Biological Invasions

According to Helen et al. [55] and Jian et al. [56], we developed a risk assessment system for IAS to evaluate the invasion risk of *S. canadensis* (Table S1). The evaluation formulas are provided in Equations (1)–(5). Risk assessment values ranging from 2.8 to 4.0 correspond to level 1 (high risk), 1.2 to 2.8 to level 2 (medium risk), and 0 to 1.2 to level 3 (low risk). The specific calculation formula is as follows:

(1) $$P=\sqrt[{4}]{P_{1}\times P_{2}\times P_{3}\times P_{4}}$$

$P_{1}$ represents the introduction and colonization risk, $P_{2}$ represents the spread risk, $P_{3}$ represents the potential hazards and impacts, and $P_{4}$ represents the control of hazards.

The calculation formula for $P_{1}$, the introduction and colonization risk, is as follows:

(2) $$P_{1}=0.3\times P_{11}+0.3\times P_{12}+0.2\times P_{13}+0.2\times P_{14}$$

$P_{11}$ represents environmental factor suitability, $P_{12}$ represents food factor suitability, $P_{13}$ represents growth and reproduction characteristics, and $P_{14}$ represents the situation of natural enemies.

The formula of $P_{2}$, the spread risk, is as follows:

(3) $$P_{2}=\sqrt[{4}]{P_{21}\times P_{22}\times P_{23}\times P_{24}}$$

$P_{21}$ represents distribution, $P_{22}$ represents existing management measures, $P_{23}$ represents spread capacity, and $P_{24}$ represents the suitable habitat range.

The calculation formula for $P_{3}$, the potential hazards and impacts, is as follows:

(4) $$P_{3}=\max\left(P_{31},P_{32},P_{33}\right)$$

$P_{31}$ represents the impact on socio-economic factors, $P_{32}$ represents the impact on the ecological environment, and $P_{33}$ represents the importance of the affected targets.

$P_{4}$, the hazard control, is calculated as follows:

(5) $$P_{4}=\left(P_{41}+P_{42}+P_{43}\right)∕3$$

$P_{41}$ represents the difficulty of identification and verification, $P_{42}$ represents the difficulty of monitoring and surveying, and $P_{43}$ represents the difficulty of control and management.

#### 4.2.4. Invasion Mechanism Analysis

To reveal the invasion mechanisms of *S. canadensis*, our study followed the method of Kreft et al. [57], and first calculated the ecological importance value of *S. canadensis* and the Shannon–Wiener Index. Subsequently, the T test method was used to compare the growth dominance of *S. canadensis* with that of other species [58]. We then used the Generalized Linear Model (GLM) to analyze the effects of species diversity and environmental factors on *S. canadensis*. To avoid the impact of multicollinearity, we chose environmental variables and excluded those with high collinearity; we finally selected 6 variables, which were bio1, bio6, bio8, bio17, bio18, and the Shannon–Wiener Diversity Index.

#### 4.2.5. Prediction of Suitable Habitat Area for *S. canadensis*

We used the Maximum Entropy Model (MaxEnt) in Species Distribution Models (SDMs) to predict the growth areas of *S. canadensis* under different future climate scenarios [59]. This model is a quantitative approach based on niche theory, utilizing species distribution data and relevant environmental data, combined with Global Climate Models (GCMs), to study species’ environmental tolerance and map their potential distribution. Prior to prediction, climate and topographic factors were resampled using the ArcMap 10.8 software. Additionally, considering the presence of autocorrelation and multicollinearity among environmental factors, we employed a Spearman correlation analysis to identify the environmental variables (Figure S2). Factors with a correlation coefficient higher than 0.75 were modeled using the Jackknife method for single-factor modeling, determining the contribution of each factor, and the final prediction variables were selected. These variables were categorized into three types. The first category was climate factors, which included biol, bio2, bio3, bio4, bio6, bio7, bio8, bio12, bio13, biol4, bio15, bio16, bio17, bio18, and bio19. The second category was topographic factors, with only one variable: elevation (elev). The third category was land cover type.

To reduce overfitting issues in the niche model analysis, the “*kuenm*” package in the R 4.3.3 software was used to evaluate different regularization factors, and feature types were adjusted based on distribution points to obtain the optimal combination for running the MaxEnt model. We used 75% of the dataset to parameterize the model and the remaining 25% for validation. Model accuracy was evaluated using the area under receiver operator curve (AUC), which ranges from 0 to 1; higher AUC values mean a better performance of this model [60]. Finally, the probability of species presence was estimated based on presence records, and background points were randomly generated by identifying the maximum entropy distribution. Using the natural breaks method in the ArcMap software, the habitat suitability was classified into four levels: non-, low, medium, and highly suitable level. Furthermore, the total area of *S. canadensis* distribution under current and future climate conditions was estimated by multiplying the number of “presence” grid cells by their spatial resolution values.

## 5. Conclusions

Our study was based on field survey data and involved analyzing and simulating the current invasion drivers of *S. canadensis* and its suitable habitat distribution under climate change scenarios. The results show that the problem of invasive alien species in the port area was serious, and due to high-intensity human interference, the level of invasion risk was relatively high. Biodiversity and the lowest temperature (especially that of the coldest month) were the key drivers of the invasion of *S. canadensis*. A highly diverse plant community could inhibit the invasion of *S. canadensis*. Meanwhile, *S. canadensis* was sensitive to low temperatures and had a distinct low-temperature limiting threshold. Under three global climate warming scenarios, the suitable habitat area of *S. canadensis* continued to increase, reaching its maximum under the medium greenhouse gas emission scenario in the 2050s. Our results provide scientific evidence for assessing and managing the risk of invasive plants, offering significant practical theoretical value for guiding source control of invasive species.

## Figures and Tables

**Table 1:** Vegetation community types in the study area.

	Community Type	Companion Species	Number of Plots	Coverage (Mean ± SD %) *	Height (Mean ± SD cm) *
1	*Solidago canadensis*	*Setaria viridis*, *Lactuca indica*, *Vicia sepium*, *Artemisia indica*	166	34.7 ± 19	64.5 ± 50.4
2	*Veronica persica*	*Geranium carolinianum*, *Vicia sepium*, *Stellaria media*	81	20.2 ± 16.9	7.1 ± 5.1
3	*Galium spurium*	*Veronica persica*, *Solidago canadensis*, *Artemisia indica*	60	12.50 ± 8.9	9.9 ± 17.3
4	*Alternanthera philoxeroides*	*Solidago canadensis*, *Erigeron canadensis*	45	32.50 ± 20.6	25.1 ± 13.3
5	*Artemisia indica*	*Veronica persica*, *Erigeron sumatrensis*, *Trifolium repens*	45	12.70 ± 5.9	6.2 ± 14.1
6	*Setaria viridis*	*Symphyotrichum subulatum*, *Solidago canadensis*, *Anthriscus sylvestris*	41	15.0 ± 5.9	72.4 ± 30.3
7	*Oxalis corniculata*	*Solidago canadensis*, *Stellaria media*	36	8.20 ± 3.8	9.4 ± 6.2
8	*Symphyotrichum subulatum*	*Artemisia argyi*, *Erigeron canadensis*	29	15.10 ± 8.6	67.1 ± 41.3
9	*Spartina alterniflora*	*Phragmites australis*	26	50.80 ± 28.2	75.7 ± 42.2
10	*Digitaria sanguinalis*	*Solidago canadensis*, *Symphyotrichum subulatum*, *Humulus scandens*	24	20.80 ± 13.2	41.2 ± 19.7
11	*Geranium carolinianum*	*Stellaria media*, *Cerastium glomeratum*	22	16.90 ± 7.8	8.4 ± 8.6
12	*Erigeron canadensis*	*Oxalis corniculata*, *Pseudognaphalium affine*	20	28.80 ± 12.5	55.6 ± 50.5
		Total number of plots	595		

* The coverage and height refer to those of the dominant species.

**Table 2:** Climate data.

Type	Factors	Description
Climate data	bio1	Average temperature for the year
	bio2	Mean daily temperature variation
	bio3	The consistency of temperature (bio2/bio7) (×100)
	bio4	Seasonal temperature variation
	bio5	Highest temperature in the warmest month
	bio6	Minimum temperature of the coldest month
	bio7	Annual temperature variation (bio5-bio6)
	bio8	Average temperature of the wettest season
	bio9	Temperature average of the driest season
	bio10	Temperature average of the warmest season
	bio11	Temperature average of the coldest season
	bio12	Total precipitation for the year
	bio13	Total precipitation in the wettest month
	bio14	Total precipitation in the driest month
	bio15	Seasonal variation in precipitation (coefficient of variation)
	bio16	Total precipitation in the wettest season
	bio17	Precipitation during the driest season
	bio18	Total precipitation in the warmest season
	bio19	Total precipitation in the coldest season

## Data Availability

The data presented in this study are available upon request from the corresponding authors.

## Supplementary Materials

The following supporting information can be downloaded at: https://www.mdpi.com/article/10.3390/plants14101546/s1, Table S1: Table of risk assessment index system of invasive alien species; Figure S1: ROC curve validation of the potential distribution prediction results for *Solidago canadensis*.; Figure S2: The results of correlation analysis of climate factors.
