# Supplementary material for: Invasion Status, Mechanisms, and Future Distribution Prediction of Solidago canadensis in the Trade Port Region: A Case Study of Ningbo Port, China
Source: Plants (Basel). 2025 May 21;14(10):1546. doi: 10.3390/plants14101546 (PMC12115084; doi:10.3390/plants14101546)
Supplement: Supplementary file 1 [file plants-14-01546-s001.zip › plants-3613138-supplementary.pdf]

# Supplementary Materials

## Invasion Status, Mechanisms, and Future Distribution Prediction of *Solidago canadensis* in the Trade Port Region: A Case Study of Ningbo Port, China

**Xu Luo [1,2]*, Sixiao Shen[1,2], Ke Liao[1], Saiqiang Li[1], Qinqin Pan[1], Jiahao Ma[1], Weiqiang Li[1], Xiaodong Yang [1,2]***

1 Ningbo University Donghai Academy, Zhejiang Ocean Development Think Tank Alliance, Ningbo 315211, China; 2211420021@nbu.edu.cn (S.S.); 2111073012@nbu.edu.cn (K.L.); 2211420010@nbu.edu.cn (S.L.); 2211420052@nbu.edu.cn (Q.P.) ; 2311110180@nbu.edu.cn (M.J.); 2311110174@nbu.edu.cn (W.L.).

2 Department of Geography & Spatial Information Techniques, Ningbo University, Ningbo 315211, China luoxu@nbu.edu.cn (X.L.); yangxiaodong@nbu.edu.cn (X.Y.).

***Correspondence:** luoxu@nbu.edu.cn; yangxiaodong@nbu.edu.cn; Tel.: +86-574-8760-9522

**Table S1.** Table of risk assessment index system of invasive alien species

| Criteria Layer | Indicator Layer | Specific Evaluation Indicators | Scoring |
|---|---|---|---|
| P1 Introduction and colonization risk | P11 Environmental factor suitability | Suitability of the climate and water environment in the evaluation area is low（0-1）;<br>Suitability of the climate and water environment in the evaluation area is moderate （0-3）;<br>Suitability of the climate and water environment in the evaluation area is high （3-4）. | |
| | P12 Food factor suitability | The suitability of animal food sources/insects, microbial hosts/soil for plant survival in the evaluation area is low；<br>The suitability of animal food sources/insects, microbial hosts/soil for plant survival in the evaluation area is moderate (1-3);<br>The suitability of animal food sources/insects, microbial hosts/soil for plant survival in the evaluation area is high (3-4). | |
| | P13 Growth and reproductive characteristics | Weak reproductive capacity (0-1);<br>Moderate reproductive capacity (1-3);<br>Strong reproductive capacity (3-4). | |
| | P14Natural enemy situation | Strong natural enemy control (0-1);<br>No effective natural enemies (3-4);<br>Other values between the two, scored based on the specific situation of natural enemies (1-3). | |
| P2 Spread risk | P21 Distribution | Distribution range<br>< 2% (0-1);<br>2%-20% (1-3);<br>>20% (3-4). | |
| | P22 Existing management measures | No corresponding management measures or strategies (0-1);<br>Listed as a quarantine or key management target at the provincial level (1-3);<br>Listed as a quarantine or key management target at the national level (3-4). | |
| | P23 Spread capacity | Can only spread short distances through natural means or migration (0-1);<br>Can only spread through human activities (1-3);<br>Can spread both through natural means and via human-assisted methods such as biological carriers, transport vehicles, or packaging (3-4). | |

**Continued table S1.** Table of risk assessment index system of invasive alien species

| Criteria Layer | Indicator Layer | Specific Evaluation Indicators | Scoring |
|---|---|---|---|
| | P24 Suitable habitat range | The proportion of suitable habitat area within the evaluation area:<br>< 2% (0-1)；<br>2%-35% (1-3)；<br>＞35% (3-4). | |
| P3 Potential hazards and impacts | P31 Impact on socio-economics | No significant economic loss caused (0-1);<br>Has caused considerable economic loss to the local area (1-3);<br>Has caused significant economic loss in the distribution area (3-4). | |
| | P32 Impact on ecological environment | Affects only one type of ecosystem, such as forest, grassland, or wetland (0-1);<br>Affects two types of ecosystems, such as forest, grassland, or wetland (1-3);<br>Affects three types of ecosystems, such as forest, grassland, or wetland (3-4). | |
| | P33 Importance of the effected targets | The affected targets have low economic value, with little impact on society and the ecological environment (0-1);<br>The affected targets have moderate economic value, with average impact on society and the ecological environment (1-3);<br>The affected targets have high economic value, with significant impact on society and the ecological environment (3-4). | |
| P4 Hazard control | P41 Difficulty of identification and verification | Reliable and fast testing methods (0-1);<br>Existing testing methods are unreliable and time-consuming (3-4);<br>Other values between the two, scored based on the specific species (1-3). | |
| | P42 Difficulty of monitoring and surveying | Monitoring methods are simple and effective for tracking population dynamics and trends of harm (0-1);<br>Monitoring and surveying are difficult (3-4);<br>Other values between the two, scored based on the specific species (1-3). | |
| | P43 Difficulty of control and management | Existing control methods are simple and effective, with a control rate of over 85% (0-1);<br>Existing control methods have a moderate control rate, between 30%-85% (1-3);<br>Existing control methods are complex and have a low control rate, below 30% (3-4). | |

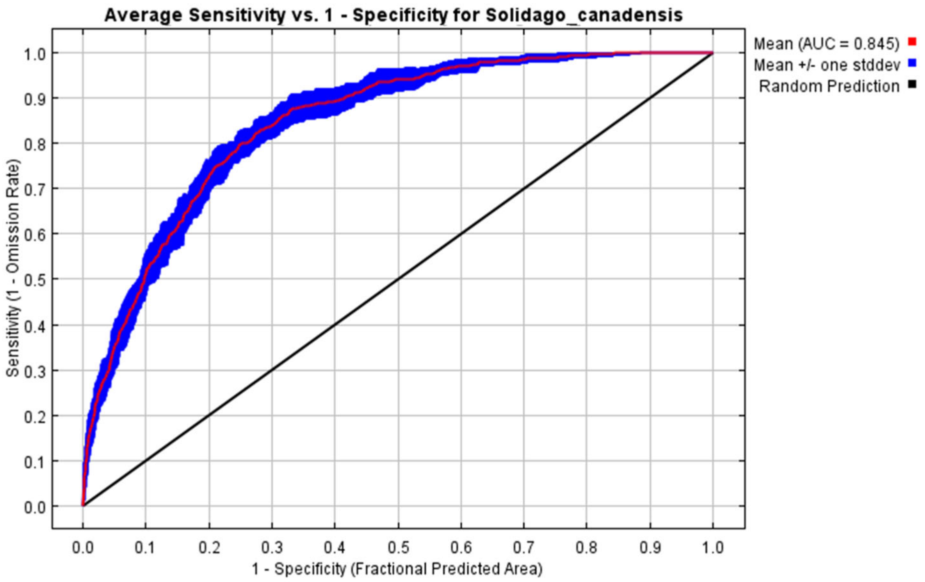


**Figure S1.** ROC curve validation of the potential distribution prediction results for *Solidago canadensis*.

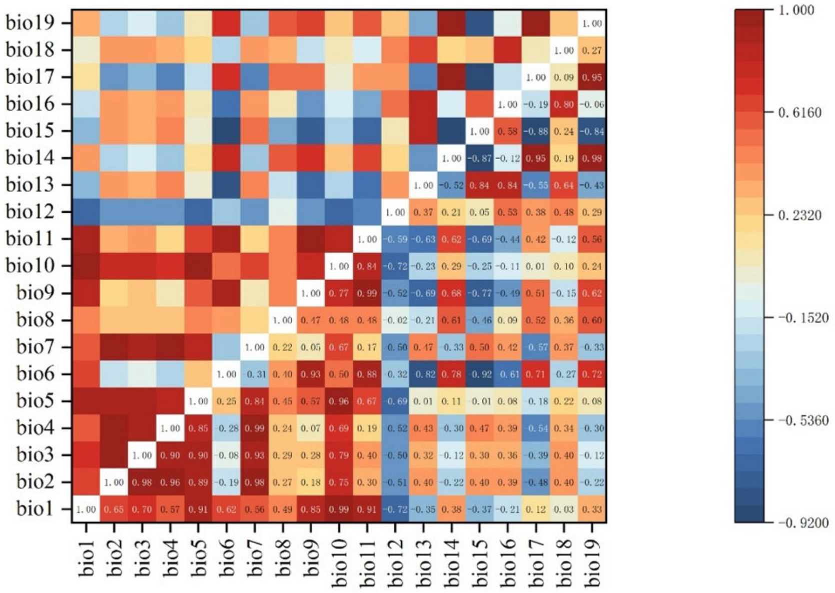


**Figure S2.** The results of correlation analysis of climate factors. The impact factors are respectively Average temperature for the year (biol), Mean daily temperature variation (bio2), The consistency of temperature (bio3), Seasonal temperature variation(bio4), Highest temperature in the warmest month (bio5), Minimum temperature of coldest month (bio6), Annual temperature variation (bio7), Average temperature of the wettest season (bio8), Temperature average of the driest season (bio9), Temperature average of the warmest season (bio10), Temperature average of the coldest season (biol1), Total precipitation for the year (bio12), Total precipitation in the wettest month (bio13), Total precipitation in the driest month (biol4), Seasonal variation in precipitation (bio15), Total precipitation in the wettest season (bio16), Precipitation during the driest season (bio17), Total precipitation in the warmest season (bio18), Total precipitation in the coldest season (bio19).
